# Solitary Endobronchial Papilloma with Malignant Transformation and Concomitant TB Infection: Case Report and Literature Review

**DOI:** 10.1155/2017/1606432

**Published:** 2017-02-08

**Authors:** Mohammed Al Ghobain

**Affiliations:** Department of Medicine, College of Medicine, King Saud bin Abdulaziz University for Health Sciences, Riyadh, Saudi Arabia

## Abstract

We are reporting a case of solitary endobronchial papilloma located in posterior segment of the left upper lobe of the lung with malignant transformation and negative human papilloma virus (HPV) strains in a 40-year-old Saudi nonsmoker man. The patient had a concomitant tuberculosis (TB) infection. The patient received appropriate treatment in the form of anti-TB medication and surgical resection of the squamous cell carcinoma followed by chemotherapy. There was no evidence of tumor recurrence, resulting in a complete cure. We are reporting the case as well as a literature review related to the topic.

## 1. Background

Solitary endobronchial papilloma is a rare papilloma entity which is usually associated with human papilloma virus (HPV) infection and occurring in middle-aged smokers. It is usually classified into three categories: squamous cell papilloma, glandular papilloma, and mixed squamous cell and glandular papilloma. Malignant transformation is rare but has been reported. We are reporting a case of solitary endobronchial papilloma located in the posterior segment of left upper lobe of the lung with malignant transformation and negative for HPV strains.

## 2. Case Presentation

A 42-year-old nonsmoking Saudi man with a history of type 1 diabetes was referred to our institution for investigation of a chronic cough and mild exertional dyspnea. He denied a history of hemoptysis, weight loss, night sweats, or change in appetite. A physical examination revealed a temperature of 37.0°C, respiratory rate of 20 breaths/min, heart rate of 70 beats/min, blood pressure of 110/70 mmHg and oxygen saturation of 96% on room air. A chest examination was entirely normal. There was no lymphadenopathy. The rest of examination was unremarkable. A complete blood count, liver function test, erythrocyte sedimentation rate and C-reactive protein were within normal limits.

**Figure 1:**
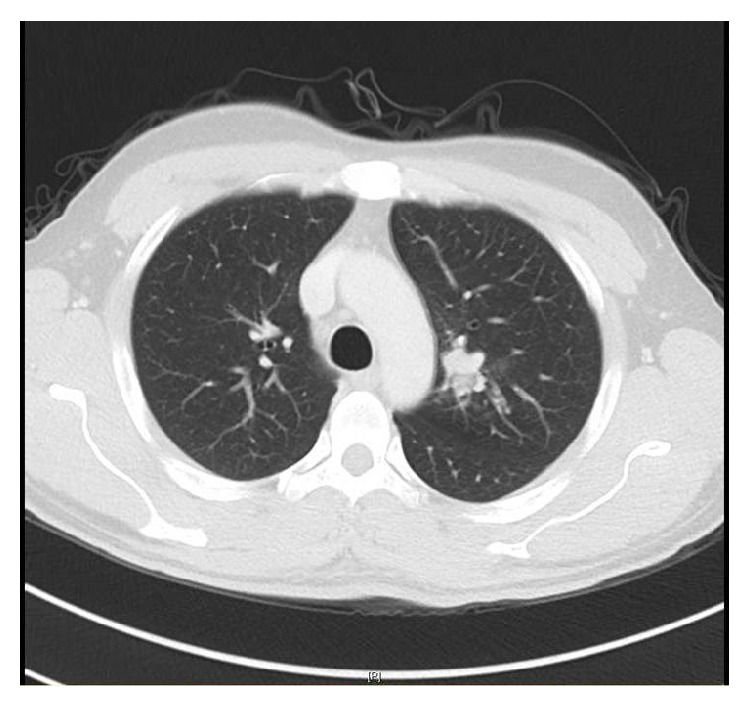
The Computed Tomography (CT) scan showed a lobulated round nodule in the left upper lobe adjacent to the bronchus measuring 1.8 × 1.6 cm with an absence of significant lymph nodes. It is associated with a focal area of ground glass opacity and mild dilatation with mucus plugging.

**Figure 2:**
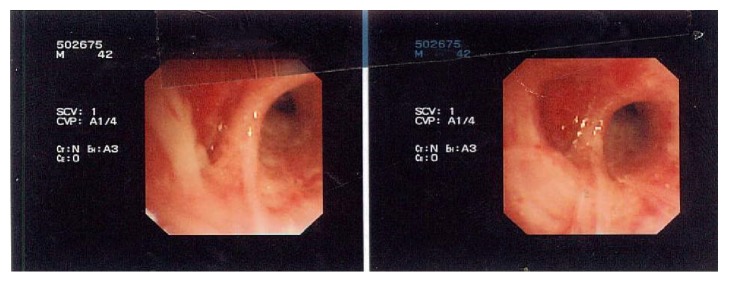
The flexible bronchoscopy revealed a swollen endobronchial lesion at the apical posterior segment of left upper lobe.

**Figure 3:**
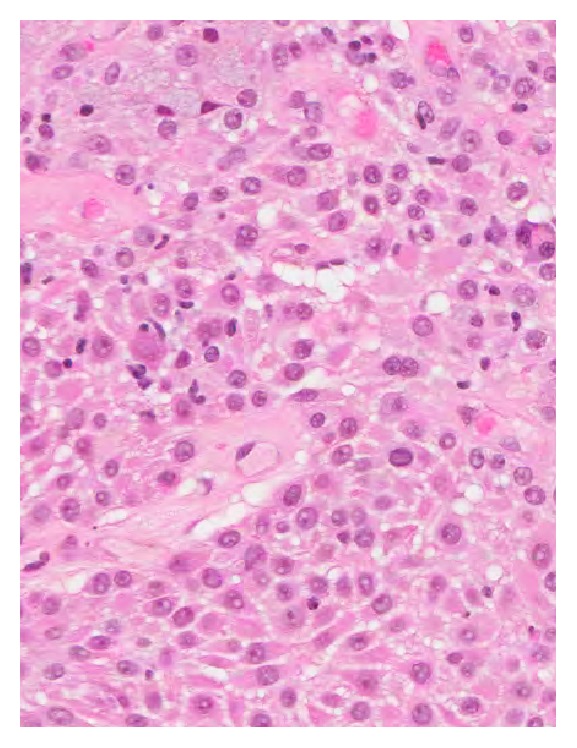
Histopathology shows benign squamous cells consistent with the diagnosis of squamous papilloma. Negative for dysplasia, malignancy, or granuloma.

**Figure 4:**
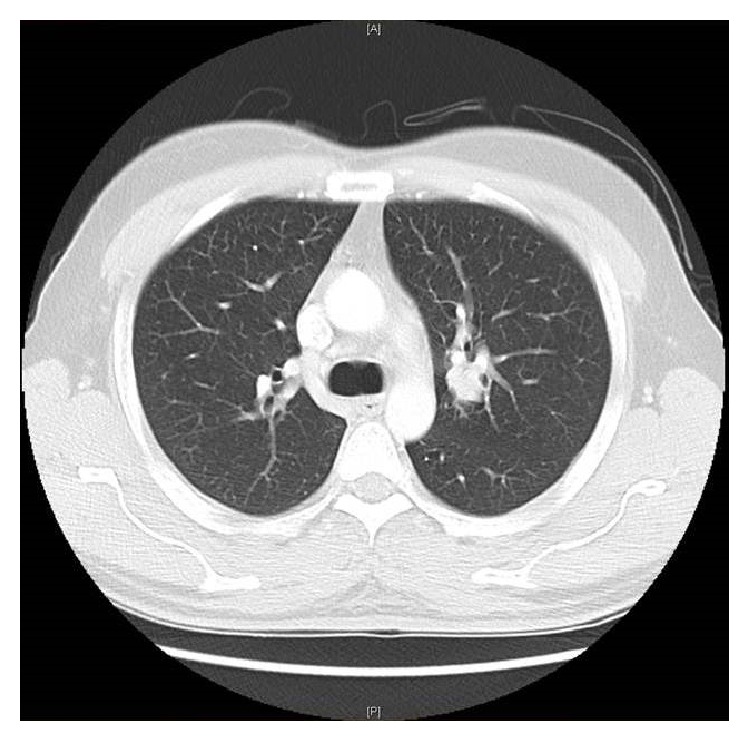
Follow-up CT chest redemonstrated the same lobulated round mass in the left upper lobe which is almost stable from the previous study. The mass is surrounded by ground glass opacity which is almost stable from the previous study.

**Figure 5:**
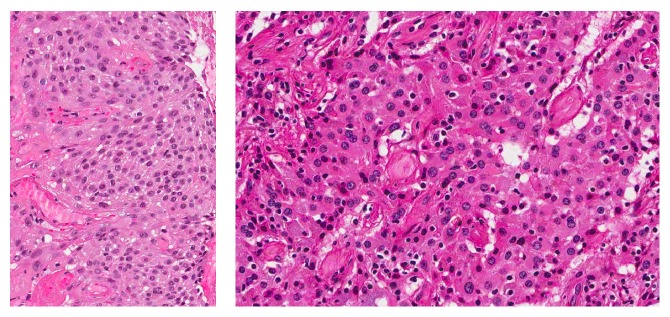
Histopathology shows a polyp lined with malignant squamous epithelium consistent with the diagnosis of well-differentiated squamous cell carcinoma.

The chest-X-ray was unremarkable. The Computed Tomography (CT) scan revealed a lobulated round nodule in the left upper lobe adjacent to the bronchus, measuring 1.8 × 1.6 cm with an absence of significant lymph nodes (Figure 1). Flexible bronchoscopy revealed a swollen endobronchial lesion at the apical posterior segment of left upper lobe of the lung (Figure 2). Cytology and brushing were negative for malignant cells. Acid fast bacilli and MT-PCR were positive and later, the* Mycobacterium tuberculosis* culture was also positive. Multiple biopsies were taken from the endobronchial lesion and histopathology was consistent with the diagnosis of squamous papilloma (Figure 3). There was no evidence of dysplasia, malignancy, or granuloma in all biopsies taken from the endobronchial lesion. HPV in situ hybridization was negative. The patient completed a 6-month course of anti-TB treatment. A follow-up CT chest redemonstrated the same lobulated round nodule in the left upper lobe, almost stable from the previous study (Figure 4). Repeated flexible bronchoscopy showed the same findings. Multiple transbronchial biopsies were consistent with the diagnosis of well-differentiated squamous cell carcinoma (Figure 5). The patient had a left upper lobectomy and biopsies of the mediastinal lymph nodes did not reveal any malignancy or granulomas (T3N0M0). The patient was treated with 6 cycles of chemotherapy and he was considered cured. He underwent a surveillance program with no evidence of recurrence after three years of follow-up.

## 3. Discussion

We are reporting a case of solitary endobronchial papilloma with malignant transformation and concomitant TB infection in a middle-aged, nonsmoking Saudi man. Our case is worth reporting considering the rarity of the solitary endobronchial papilloma. What is remarkable is the uncommon malignant transformation of this rare condition which is usually associated with smoking and positive HPV. In addition, the patient had a concomitant TB infection which we think has no relation with the endobronchial papilloma and considered as an accidental finding as the patient has no symptoms suggestive of TB infection. Up to our knowledge, there is no data to describe the case of endobronchial papilloma and concomitant TB infection. It is, however, worth further investigation. Our patient received appropriate treatment in the form of anti-TB medication and surgical resection of the squamous cell carcinoma followed by chemotherapy with no evidence of tumor recurrence resulting eventually in a complete cure.

Solitary papilloma is commonly found in genitalia or oral cavity but it is very unusual to be found in the airways as in our patient. Solitary endobronchial papilloma is a rare papilloma representing only 0.5% of all types of papilloma and 0.3% of all lung tumors [1].

Because of the rarity of solitary endobronchial papilloma, little is known about the epidemiology in terms of gender, age distribution, and the relationship with environmental factors. A review of 41 papilloma cases found 76% to occur in men [2]. Additional studies have investigated the potential association with HPV. In squamous papilloma, HPV DNA is estimated in 50% of tumors [2]. Among all different types of HPV, each is associated with different malignant potential and symptom severity. HPV types 6 and 11 are the most common strains identified in cases of solitary tracheobronchial papilloma and associated with a low risk of malignant transformation. In addition, HPV types 16 and 18, occasionally in combination with types 31, 33, or 35, are associated with a higher risk of malignant transformation [3].

Solitary endobronchial papilloma is usually classified into 3 types: squamous papilloma (in smokers and associated with HPV), glandular papilloma, and mixed type. Two multiple papillomas of the tracheobronchial tree with malignant transformation have been first reported by DiMarco et al. [4]. Malignant transformation was found in 5% of cases of papilloma and usually associated with squamous papilloma, smokers, HPV types 16 and 18, and the patient older than 40 years of age [3]. In our case, however, the patient was a nonsmoker and the HPV strains were negative. Solitary endobronchial papilloma can easily be mistaken for malignancy. An-Ning Feng et al. [5] reported two cases of solitary endobronchial papillomas; one was a squamous cell papilloma providing a false impression of interstitial microinvasion. The other was a mixed squamous cell and glandular papilloma with a gross appearance of massive lipid pneumonia, which focally resembles adenocarcinoma with a lepidic-like pattern on histological examination. Tryfon et al. [6] reported 32 cases of solitary endobronchial papillomas between 1986 and 2008 in Greece; the estimated incidence was 3.95 cases/100,000 patients/year. It occurs more commonly in men (ratio 3 : 1). Squamous papillomas occur commonly during the fifth decade of life, glandular papillomas predominate in the sixth decade, and the distribution of mixed type papillomas is from the third to the sixth decade of life. In the Tryfon et al. review, 5 of 32 patients (15.65%) presented with malignant degeneration; two of the patients developed squamous cell carcinoma, one small cell carcinoma, one glandular carcinoma, and one low-grade carcinoma [6].

In our case, there is a possibility of the existence of squamous carcinoma at the initial presentation of the patient. However, multiple biopsies were taken from the endobronchial lesion and histopathology was consistent with squamous papilloma. There was no evidence of malignancy or granuloma in all biopsies taken from the endobronchial lesion.

Solitary endobronchial papilloma has a variety of clinical presentations. It can be without symptoms and discovered incidentally. If symptoms occurred, it includes cough, dyspnea, hemoptysis, wheezing or asthma-like symptoms, recurrent pneumonia, lobar collapse, atelectasis, air trapping, postobstructive infections, and bronchiectasis as a complication of bronchial obstruction [7]. Paganin et al. [8] describe a case of solitary mixed papilloma presenting with hemoptysis and located in the proximal part of the main right bronchus and treated with endobronchial electrocautery.

Radiological features are variable and not diagnostic for endobronchial papilloma. Features may include a shadow, infiltrates, mass, pulmonary nodule or postobstructive atelectasis, and bronchiectasis.

In conclusion, solitary endobronchial papilloma is a rare tumor. Clinical and radiological presentations are not diagnostic and cannot differentiate the papilloma from other tumors. A biopsy is required to make the diagnosis and malignant transformation, though it is rare, is a potential complication which should be considered during the management of the patient.

## References

[1] Travis W. D., Colby T. V., Corrin B., Shimosato Y., Brambilla E. (1999). *Histological Typing of Lung and Pleural Tumours*.

[2] Flieder D. B., Koss M. N., Nicholson A., Sesterhenn I. A., Petras R. E., Travis W. D. (1998). Solitary pulmonary papillomas in adults: a clinicopathologic and in situ hybridization study of 14 cases combined with 27 cases in the literature. *American Journal of Surgical Pathology*.

[3] Lang T. U., Khalbuss W. E., Monaco S. E., Pantanowitz L. (2011). Solitary tracheobronchial papilloma: cytomorphology and ancillary studies with histologic correlation. *CytoJournal*.

[4] DiMarco A. F., Montenegro H., Payne C. B., Kwon K. H. (1978). Papillomas of the tracheobronchial tree with malignant degeneration. *Chest*.

[5] Feng A.-N., Wu H.-Y., Zhou Q. (2015). Solitary endobronchial papillomas with false impression of malignant transformation: report of two cases and review of the literature. *International Journal of Clinical and Experimental Pathology*.

[6] Tryfon S., Dramba V., Zoglopitis F. (2012). Solitary papillomas of the lower airways: epidemiological, clinical, and therapeutic data during a 22-year period and review of the literature. *Journal of Thoracic Oncology*.

[7] Miura H., Tsuchida T., Kawate N., Konaka C., Kato H., Ebihara Y. (1993). Asymptomatic solitary papilloma of the bronchus: review of occurrence in Japan. *European Respiratory Journal*.

[8] Paganin F., Prevot M., Noel J. B., Frejeville M., Arvin-Berod C., Bourdin A. (2009). A solitary bronchial papilloma with unusual endoscopic presentation: case study and literature review. *BMC Pulmonary Medicine*.

